# Reproducibility and Scalability of Magnetic Nanoheater Synthesis

**DOI:** 10.3390/nano11082059

**Published:** 2021-08-13

**Authors:** Jesus G. Ovejero, Alvaro Gallo-Cordova, Alejandro G. Roca, M. P. Morales, Sabino Veintemillas-Verdaguer

**Affiliations:** Department of Energy Environment and Health, Instituto de Ciencia de Materiales de Madrid, C.S.I.C., Sor Juana Inés de la Cruz 3, Cantoblanco, ES28049 Madrid, Spain; jesus.g.ovejero@csic.es (J.G.O.); alvaro.gallo@csic.es (A.G.-C.); alejandrogroca@gmail.com (A.G.R.); puerto@icmm.csic.es (M.P.M.)

**Keywords:** magnetic nanoparticles, organic decomposition, scaling up, reproducibility

## Abstract

The application of magnetic nanoparticles requires large amounts of materials of reproducible quality. This work explores the scaled-up synthesis of multi-core iron oxide nanoparticles through the use of thermal decomposition in organic media and kilograms of reagents. To this end, we check the effect of extending the high temperature step from minutes to hours. To address the intrinsic variability of the colloidal crystallization nucleation process, the experiments were repeated and analyzed statistically. Due to the simultaneity of the nuclei growth and agglomeration steps, the nanostructure of the samples produced was a combination of single- and multi-core nanoparticles. The main characteristics of the materials obtained, as well as the reaction yields, were analyzed and compared. As a general rule, yield, particle size, and reproducibility increase when the time at high temperature is prolonged. The samples obtained were ranked in terms of the reproducibility of different structural, colloidal, and magnetic features. The capability of the obtained materials to act as nanoheaters in magnetic hyperthermia was assessed, showing a strong dependence on the crystallite size (calculated by X-ray diffraction), reflecting the nanoparticle volume with a coherent magnetization reversal.

## 1. Introduction

Many applications have been envisaged for magnetic nanoparticles (MNPs) in recent decades, most of which are in the biomedical field, for example, their use as contrast agents in magnetic resonance imaging [1,2] or for cancer treatment through their capacity to generate heat under alternating magnetic fields (AMF) [3], among others. In the last decade, new applications related to biocatalysis have indicated that these MNPs not only can support enzymes or biomolecules efficiently, which can be easily separated by means of a magnetic field, but they are also able to activate them [4,5] when subjected to an AMF [6,7,8]. The local heat generation at the MNP surface and its control at the nanoscale are hot research topics in the nanotechnology field [9].

To date, the only industrial process able to produce MNPs in reasonable quantities is coprecipitation from aqueous solutions [1,10]. Unfortunately, the MNPs obtained by this method rarely have a particle size exceeding 10 nm, while most of the previously mentioned applications of such materials benefit from a size increase (and, therefore, magnetization per particle) above this limit. To attain this goal, the decomposition of organic precursors at high temperatures in the presence of surfactants has been widely employed since it was proposed for MNPs at the beginning of the 21st century [11,12]. This synthesis method outperforms other alternatives for the chemical production of large MNPs (>20 nm) in terms of size homogeneity, shape control, and compositional versatility [13,14]. It is common to find articles claiming the synthesis of a large quantity of nanoparticles with outstanding homogeneity and narrow size distributions; however, the experimental procedures described rarely produce more than a gram of product [12,13,14,15,16,17], and none of them have performed a systematic study of the degree of reproducibility of such scaled-up processes. There are several factors that may lead to the irreproducibility of the scaled-up protocols, such as degradation of the reactor glass, variability of the precursors, or poor temperature control in large-volume reactors.

In this work, we present the scaled-up synthesis of multi-core MNPs by organic decomposition at a high temperature, using reactants on the order of up to kilograms and cheap commercial precursors. This has been identified as a mandatory prior step to the production of such materials at the pilot plant scale [16]. The synthesis was repeated in order to explore the reproducibility of the process in terms of chemical yield and particle size and a wide range of structural, colloidal, and magnetic properties of the products selected as quality control parameters. The maturation time at the highest temperatures was identified as a critical parameter for controlling the MNP size and microstructure, and, therefore, it was analyzed in further detail.

## 2. Materials and Methods

Magnetite nanoparticles were prepared by decomposition of iron (III) acetylacetonate 99% (Acros Organics, Geel, Belgium) in benzyl ether (99%; Acros Organics, Geel, Belgium) in the presence of oleic acid (OA; 80%; GPR Rectapur^®^, VWR, Leicestershire) and 1,2-dodecanediol 90 % (ODA; Sigma Aldrich, San Luis, MO, USA). The molar ratio Fe(acac)_3_:OA:ODA was 1:3:2, and the concentration of the iron precursor was 0.1 M. The amounts of reagents employed were Fe(acac)_3_, 35.3 g (100 mmol) equivalent to 7.7 g of magnetite; benzyl ether, 1000 g; OA 105.9 g (300 mmol); and ODA, 44.96 g (200 mmol). The price of the synthesis was kept at a minimum by choosing more affordable reagents of standard quality, which were used without previous purification. The reagent’s final volume was 10% of the reactor capacity employed in the synthesis (10 L, quartz). The low filling ratio of the reactor assured the fast heating of the mixture, using a standard 1300 W 10 L heating mantle (LabHeat^®^ KM-ME, SAF Wärmetecknik GmbH, Mörlenbach, Germany).

Aside from the temperature ramp and the stirring rate [15], many modifications must be made to scale up a process. For example, in order to have good temperature reproducibility in a large volume of viscous fluid, it is necessary to substitute the standard digital PID temperature controller (based on the temperature measurement at one point) with a delivered electrical power control. Furthermore, the substitution of the centrifugation (which is difficult for high volumes at laboratory scale) with magnetic decantation, in order to separate the product, is also relevant. Additionally, 1-octadecene was substituted by benzyl ether in order to ensure easy purification of the products. In detail, the synthetic procedure was as follows: First, the mixture of reagents was homogenized in a 2 L glass beaker using an Ultra-thurrax^®^ (Staufen, Germany) at 6000 rpm/20 min before introducing it into the reactor. Then, the mixture was overhead stirred at 100 rpm, and nitrogen (9.5 L/min) was flowed through the stirrer guide for 1.5 h (stirring and nitrogen flow were maintained throughout the process). The reactor (isolated externally with glass wool) was heated at 670 W until reaching 195 °C (1 h); then, the reflux refrigeration started, and the power was reduced to 244 W in order to maintain the temperature at approximately 200 °C for 2 h. Finally, the mixture was heated at full power (1300 W) up to the boiling temperature (~285 °C) for a variable amount of time (5, 15, 30, 60, or 120 min). Immediately after, the stirring was stopped, and the heating mantle removed in order to quench the reaction while maintaining the nitrogen flow. The following day, the product (1 L) was pumped out to a 5 L glass beaker and precipitated using a mixture of 333 mL n-hexane (96%; ACS BASIC^®^, Scharlau, Madrid, Spain) and 1000 mL ethanol (96% *v*/*v*; Pharmapur^®^, Scharlau, Madrid, Spain) settled over a 0.5 T neodymium magnet (60 mm diameter, 30 mm high) for two days in order to separate the magnetic fraction. After discarding the supernatant, the solid was washed three times with 200 mL of toluene (99.5%; EssentQ^®^, Scharlau, Madrid, Spain):ethanol 1:2 *v*/*v*, sonicated for 15 min, and magnetically separated. After the last wash, the supernatant was transparent, and the final product was dispersed with sonication in a solution of oleic acid:toluene 1:7 *v*/*v* (150 cc toluene and 20 g of oleic acid).

For comparison purposes, six samples were prepared at standard laboratory scale (60 g of reactants) in a one-liter (quartz) reactor using the same concentration of reactants, stirring rate, and temperature profile as for the up-scaled samples for a fixed boiling time of 30 min. Samples were named as OR or ORD depending on if they were scaled up or unscaled 1:20 times, respectively, followed by the experiment number.

The dispersion (80 μL) was digested with 3 mL of aqua regia at 90 °C for 20 h and analyzed for iron by using inductively coupled–optical emission spectroscopy (ICP-OES) [17]. The value of the iron concentration after sample washing was used to calculate the yield of the process. Dry product was obtained by precipitation of 3 mL of the dispersion with 10 mL of methanol, followed by washing one time with 10 mL methanol and drying at 50 °C. The samples were characterized by transmission electron microscopy (TEM, JEOL JEM 1011, Peabody, MA, USA), X-ray powder diffraction (XRD, Bruker D8 Advance, Billerica, MA, USA), thermogravimetric analysis (TG-DTA, Q600 TA Instruments, New Castle, DE 19720, USA), and magnetometry (VSM, OXFORD Instruments, Abingdon, UK); see Supplementary Materials. The capacity of the toluene dispersions to generate heat under AMF was measured after dilution to 1 mg Fe/mL in oleic acid:toluene 1:7 *v*/*v* and sonication for 15 min, applying magnetic fields of 64 mT (50 kA/m) at 92 kHz and 22 mT (18 kA/m) at 286 kHz using an AC magnetic inductor (Fives Celes 12118 M01, Lautenbach, Francia). The temperature was measured using a fiber optic thermometer (Osensa PRB-G40-02M-STM-MRI, Burnaby, BC, Canada). In order to check the stability of the dispersions in front of the magnetic field, the heating ramps were measured five times, and the average heating ramp (or the first one, in the case of an unstable sample) was considered for the SAR calculation (Figure S2). The solvodynamic size of the oleic acid:toluene 1:7 *v*/*v* dispersions was determined by photon correlation spectroscopy (PCS, Zetasizer Nano, Malvern Panalytical, Malvern, UK).

To check the reproducibility of the process, the experiments at each boiling time were repeated six times. The average results are presented as $\bar{x}$ ± z· σ/√N, where $\bar{x}$ is the average value of the property reported, σ is the standard deviation, z = 1.96 is the t-student statistic for the 95% confidence index, and N is the number of experiments (which was six in this case) [18]. The repeatability (r) of the control experimental parameters and the results were evaluated as (1 − σ/$\bar{x}$) · 100. Confusion among the standard deviation (σ) of the averages employed in the previously mentioned calculations and the standard deviation of the particle size distribution determined by TEM (σ_TEM_) must be avoided.

## 3. Results

The average experimental temperature profiles and their standard deviations obtained for each boiling time are depicted in Figure S1. The temperature profile was close to that reported previously [15], starting with an initial temperature ramp of 2.77 °C/min average, followed by a plateau at 203 °C average temperature for two hours, changing afterward to a heating temperature ramp at a maximum power of 9 °C/min average, up to boiling and finishing with the reflux stage at 286 °C. The corresponding thermal parameters and their repetitiveness are given in Table S1. The heat flux was estimated to be approximately equal for the scaled experiment and the standard one, but the energy delivered per gram of reactant was smaller in the scaled experiment (see Supplementary Materials). The results support the chosen strategy of using the power supply as an easier and more reliable control of the thermal ramp in the scaled-up synthesis. In the following subsections, the repeatability of the results of the process and their dependence on the boiling time are presented. The individual values for the yield and the characterization results of all the experiments performed in this study are presented in Table S2. Then, we discuss the average values obtained in each set of experiments at a certain boiling time and the variation among them.

### 3.1. Reaction Yield as a Function of the Boiling Time

The average yield of the reactions is presented, in Table 1, as a function of the boiling time. The main observation is that the productivity of the scaled process increased with the boiling time, up to 61–71%. It is also worth noting the low yield obtained for the unscaled reference sample (24%). Roughly, we could explain the yield increase with the boiling time as a logical consequence of prolonging the decomposition time of the reactants at the highest temperature. The low yield of the unscaled process could be attributed to the better homogeneity, smaller solvodynamic sizes, and improved colloidal stability of these samples, which were more difficult to separate with the magnet, as discussed in the following section. For each set of experiments performed under the same conditions, the interplay of both factors—that is, the boiling time and the effectiveness of the magnetic separation—justifies the important yield variations observed.

### 3.2. Evolution of the Average Particle Size and Particle Size Distribution

**Figure 1:**
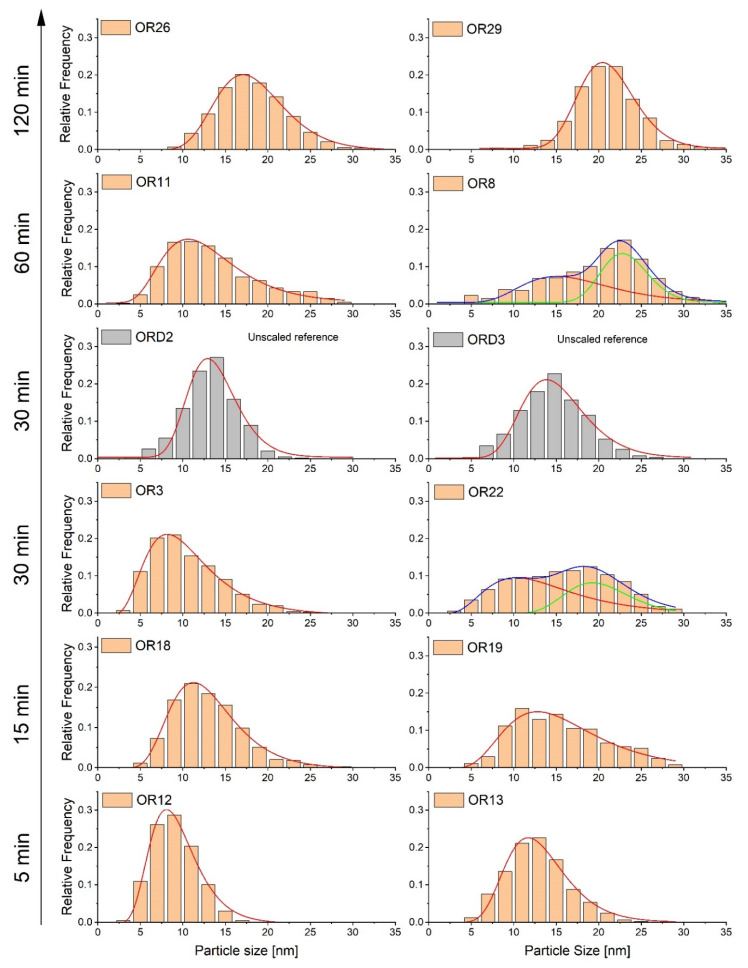
Evolution of TEM particle size distributions of two extreme scaled-up samples heated during different times at the highest temperature (5, 15, 30, 60, 120 min). For each boiling point time, two samples with the highest and lowest average size were chosen. The grey distribution corresponds to the unscaled samples, ORD2 and ORD3.

The evolutions of the average particle size and size distribution determined by TEM with boiling time show two regimes (Figure 1, Table 1). In the first regime (t = 0–30 min), the average particle size remained approximately constant, at around 13–14 nm, and was coincident with Scherrer’s size. However, the distribution becomes broader arising a second population of particles attributed to the formation of multi-core nanoparticles (Figure 1), and, consequently, the polydispersity (σ_TEM_/D_TEM_) increases up to 0.36. In the second regime, a further increase in particle size (up to 20 nm) appeared at longer boiling times from 30 to 120 min, with an important reduction in polydispersity (σ_TEM_/D_TEM_; <0.25). The highest polydispersity appeared under intermediate boiling times (0.36 for 30 min) with small variations, and, in general, it was significantly higher than the unscaled samples obtained under the same conditions. To illustrate the variability of the particle size distribution (PSD) obtained, the PSD of the two extreme average sizes (minimum and maximum) for each boiling time is presented in Figure 1. It is evident, from this figure, that the secondary PSD formed when the boiling time was increased to 30–60 min, was the cause of the increment of average particle size and polydispersity.

**Figure 2:**
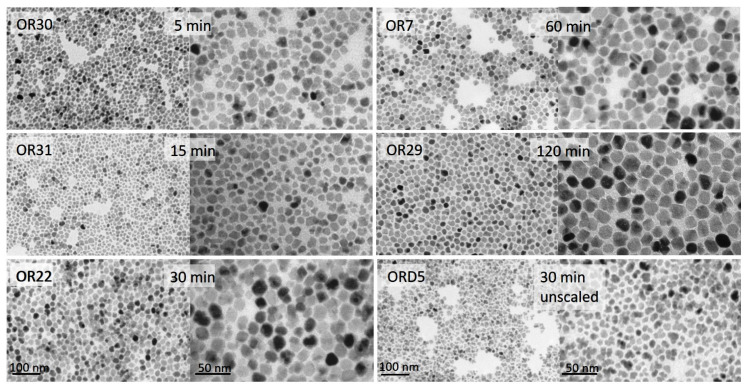
TEM images of one representative scaled-up sample heated for different times at the highest temperature and one unscaled sample (ORD5) heated for 30 min at the highest temperature.

**Figure 3:**
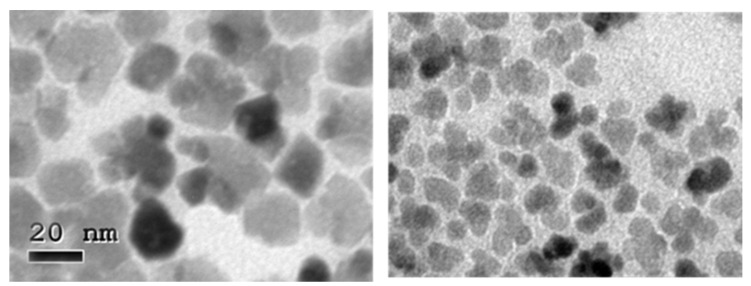
TEM images at higher magnification of aggregates of MNPs in a scaled sample (OR2, left) and in the unscaled sample (ORD6, right), both prepared with 30 min boiling time.

Representative TEM micrographs are shown in Figure 2. In these pictures, the appearance of single- as well as multi-core nanoparticles can be appreciated. The presence of such entities is responsible for the evolution of sizes and polydispersity observed. Enhancement of the aggregation (also present in the unscaled samples; see Figure 3) was the most important new phenomenon observed in our scaled experiments by this point.

A single phase, inverse spinel structure similar to magnetite/maghemite is obtained in all cases as shown in Figure S3. Besides, the constancy of Scherrer’s size through this study for the scaled samples (13–14 nm) is worth noting, as it is slightly smaller for the unscaled samples (12.3 nm). Although a fine crystal size determination requires advanced Rietveld refinement that goes beyond the scope of the present article [19], this result strongly suggests that the multi-core nanostructure obtained here presented less internal core orientation than that observed in the nanoflowers obtained by a polyol process, despite their similar appearance [20,21]. The lower organic content observed in the scaled samples, with respect to the unscaled ones at the same boiling time, suggests that the surfactants present in the media were more unstable in the scaled experiments. It is probable that surfactant deprivation may produce a deficient capping of the crystals grown, leading to the aggregation of the cores and, thus, the formation of multi-core structures as clearly shown in the TEM images at higher magnifications (Figure 3).

DC magnetization curves for all samples in powder form were measured at room temperature (Figure S4). They showed a nearly superparamagnetic behavior at this temperature and saturation magnetization values of 63–67 Am^2^/Kg before organic content correction (Table 1), that is 98–104 Am^2^/Kg_Fe_, which is slightly below the expected value for bulk maghemite (M_S_ = 118 Am^2^/kg_Fe_). Susceptibility is in all cases very high, around 900 Am^2^ Kg^−1^ T^−1^, but slightly smaller than the value for the unscaled sample, 1200 Am^2^ Kg^−1^ T^−1^, probably due to a smaller aggregation degree.

### 3.3. Solvodynamic Size and Magnetothermia of the As-Synthesized Suspensions

In Table 2, we present the colloidal properties of the samples produced, including average values of the solvodynamic size and polydispersity indices (PDIs). The presence of a certain degree of aggregation in the scaled samples was clear, arising mainly from the magnetic purification after the synthesis, and which increased progressively with the boiling time (Data of number and volume distribution are included in Figure S5). Those aggregates persisted through the several sonication processes that the samples underwent during cleaning at high concentration (>10 mg Fe/mL), as well as at high dilution (0.1 mg Fe/mL) during the preparation of the colloids for the PCS examination. Interestingly, the solvodynamic size of the unscaled sample is significantly smaller than that for the up-scale samples, probably due to the degradation of the surfactants in the upscale synthesis.

The heating efficiency of the samples was evaluated by measuring the SAR under two different field conditions—high frequency and low field and low frequency and high field (286 kHz/18 kAm^−1^ and 92 kHz/50 kAm^−1^, Figure S6)—at 1 mg Fe/mL in order to reduce the concentration effects of interacting MNPs, such as the SAR variations from 85 to 125 W/g reported for 20 nm MNPs, as the concentration increased from 4 to 15 mg Fe/mL under similar field conditions (105 kHz, 40 kAm^−1^) [22].

SAR values of the scaled samples were approximately constant with boiling time, independent of the TEM particle size and the PCS solvodynamic size (Table 1 and Table 2), and they were slightly lower for the unscaled samples. These results point to the fact that the SAR values, as well as the saturation magnetization and magnetic susceptibility, were sensitive mainly to Scherrer’s size, which was constant for all the scaled samples and slightly smaller for the unscaled ones.

The independence of SAR, with respect to the solvodynamic size of the samples, indicated that the main mechanism of magnetic relaxation for these MNPs under such field conditions is Neel relaxation instead of Brownian rotation. This mechanism is expected to be dominant in magnetite nanoparticles smaller than 15 nm [23], which is just above the 13 nm mean Scherrer’s size measured by XRD (Table 1). The SAR values obtained for the multi-core 19 and 22 nm MNPs were smaller than those reported previously for multi-core particles prepared in polyol [21] at similar low frequencies, and they responded to the alternating magnetic field as if they were much smaller. We understand that this is the consequence of the magnetic independence of the particles in the multi-core structure mentioned previously (Figure 3). The outstanding SAR values reported for multi-core nanostructures have been attributed to the exchange coupling established between adjacent cores with epitaxial interfaces [24,25]. Actually, in this kind of system, the crystal size obtained from the XRD results was larger than the core size due to the coherent diffraction of the comprising cores.

In general, the effect of the nanoparticle size on the heating capacity of a suspension of MNPs under an alternating magnetic field is expected to increase as particle size increases, although many other factors can mask this result due to collective behavior forming chains, for example, and poor colloidal stability, which can lead to huge errors due to the precipitation of nanoparticles (Figure S2). Such sample degradation issues complicate the comparison between different MNPs and equipment [26]; however, experimental data in the literature have shown SAR values between 20 and 65 W/g (40 kAm^−1^, 77 kHz) for samples synthesized by thermal decomposition in 1-octadecene, with the same mean size (14 nm) but different size distribution [27]. The highest SAR was closer to the data reported here under similar field conditions and was associated with the sample with the narrowest size distribution. When increasing the frequency, similar SAR values as those found here (between 100 and 200 W/g) have been reported for 15–20 nm particles synthesized by thermal decomposition at a 50 mL scale [28]. These values confirm the competitiveness of the obtained samples as magnetic nanoheaters.

### 3.4. Reproducibility

**Figure 4:**
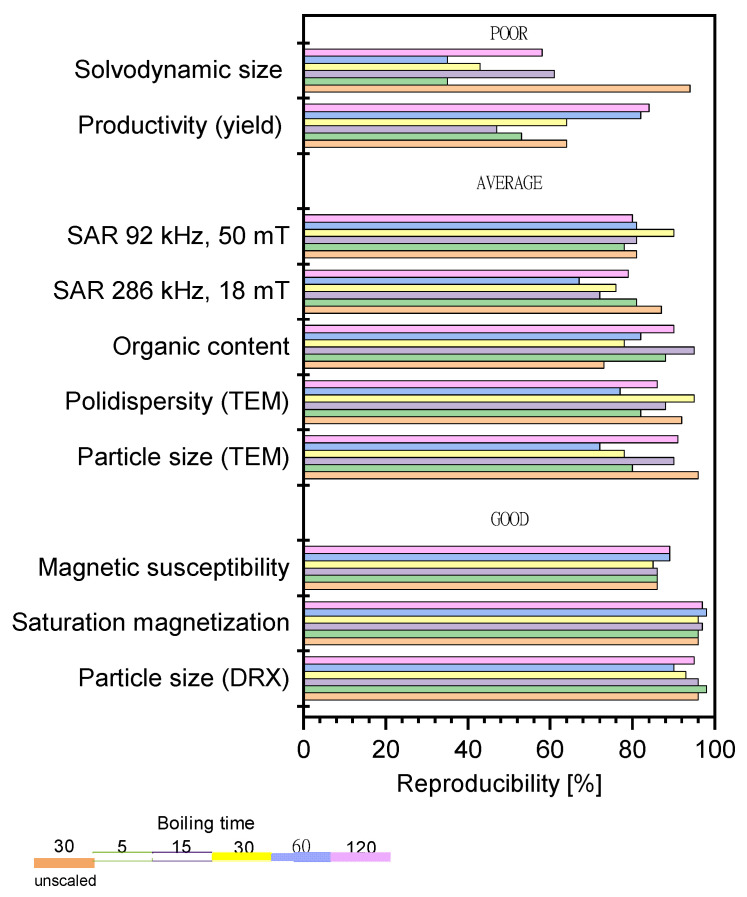
Reproducibility of the process and sample characteristics, as well as their dependence on the boiling time. The control parameters have been broadly classified in terms of reproducibility as poor (<60%), average (60–80%), and good (>80%).

In Figure 4, we plot the reproducibility percentage of a selection of quality control parameters, measured for the different batches under each set of experimental conditions.

The agreement between the results obtained for the different batches under the same experimental conditions spanned a wide interval (50–90%) for low and intermediate boiling times and tended to cluster around 75% when the boiling time increased up to 2 h, which is presumably when the system reaches an equilibrium state. Therefore, we conclude that this value of 75% represents the average reproducibility of the scaled-up production of MNPs attained in the present study. It is interesting to observe the differences in repeatability among the parameters measured. Very good reproducibility was determined for Scherrer’s size and the magnetic properties. These were the most reproducible properties both in scaled and unscaled experiments, independent of the boiling time. Reasonably good reproducibility (>70%) was obtained for the chemical composition, TEM particle size, and SAR values. The reproducibility of the TEM measurements for the scaled samples was smaller than for the unscaled ones, likely due to the influence of variability of the aggregation process, which governs the final particle size of the MNPs produced at high scale. An opposite tendency was observed for the organic content, which was more reproducible for the scaled than unscaled samples. SAR values showed greater reproducibility at low frequency, independent of the boiling time. At high frequency, we found lower repeatability, which was dependent on the boiling time—we noted that it was higher for short and long boiling times (when the variability of the aggregation appeared to be less important). By far, the least reproducible parameters were the yield of the reaction and the solvodynamic size for both scaled and unscaled samples. The yield of the reaction was mainly influenced by the effectiveness of the magnetic separation, depending on the particle size and the colloidal stability of the suspensions. The solvodynamic size was more affected by scaling up due to its high sensitivity to the polydispersity of the particle size and the organic content. This can be explained by the specific features of the synthesis method. The high boiling temperature required for precursor decomposition compromised the stability of the surfactants and, consequently, reduced the colloidal stability of the final product. This is especially true for the scaled synthesis, in which the degradation of oleic capping led to the formation of multi-core nanostructures of a larger size. These issues could be easily addressed with a further step of polymer coating, magnetic selection and stabilization.

### 3.5. Production Cost

By synthesizing MNPs via thermal decomposition method, it was possible to scale up the productivity at laboratory scale by about 50 times. Therefore, due to the six-fold increment of reactants, the increase from 0.06 to 5 g of product seems feasible. However, it is interesting to estimate the exact benefits provided by large-scale production. In addition to the possibility of industrialization with mass production, there are several factors to be considered in terms of time and economy. Some of these are the following: (1) batch-to-batch reproducibility, (2) lower production costs, (3) economy of labor (per unit labor cost is reduced), (4) less expenses in administration and distribution, and (5) possible utilization of by-products. In general, scaling up processes is the best way to produce knowledge and transfer ideas into fruitful commercial implementations [29]. Under these considerations, the total production cost was estimated for the unscaled and large-scale synthesis in Table 3. These numbers clearly evidence that it is possible to diminish the final production cost by increasing the quantities of reagents, thus reducing the time and energy costs of the process.

One of the examples of a scaled synthesis of iron oxide nanoparticles has been performed by Park et al., using a thermal decomposition approach similar to the one reported here [10]. However, this synthesis required the preparation of an oleate precursor, which introduces an important degree of variability in the synthesis [30]. Other approaches, such as bacterial biotransformation [31] or coprecipitation [32] of iron salts, are suitable for kg-scale production of MNPs but present intrinsic polydispersity, thus reducing their applicability in high-tech industries. Microwave-assisted heating, on the other hand, is an interesting solution to produce a large amount of MNPs; however, the sizes are generally smaller than 15 nm [33]. It is worth mentioning the big effort invested at present in the design of continuous-flow and microfluidic production methods, which offer great advantages in terms of continuous production and the reduction of human intervention [34,35]. In comparative terms, and under determinate conditions, the proposed synthesis represents a cost-effective alternative in terms of monodispersity and especially reproducibility.

## 4. Conclusions

Scaling up the synthesis of magnetic nanoparticles by thermal decomposition in organic media under the conditions reported in this work presented similar reproducibility, on average, as the unscaled synthesis, as long as the reaction is prolonged for long boiling periods. The scaled process produces more agglomerated colloids, likely due to difficulties in homogenization of the system. The principal conclusion of this work is that the scaled-up production of nanoparticles does not necessarily work well for those experimental conditions that do so at the low laboratory scale. For example, in our setting, 15 min was the optimum boiling time when scaling up the production of 14 nm nanoparticles, while 120 min was the optimum for the production of 19 nm nanoparticles. Furthermore, it was possible to obtain an impressive reproducibility of Scherrer’s size despite the fact that, in the scaled-up process, there was particle agglomeration.

It is important to acknowledge that here we present a breakthrough in the scalability and reproducibility of multi-core iron oxide magnetic nanoparticles prepared by a thermal decomposition method in organic media, obtaining 5 g per batch (70% yield). This is certainly a step toward the commercial use of these nanoparticles in different applications, such as biocatalysis, where high production volumes are an essential prerequisite for their implementation.

## Figures and Tables

**Table 1:** Average values of the six repetitions are reported here for the yield, particle size and polydispersity (TEM), crystallite size (XRD), organic content (TG-ATD), and magnetic properties at RT (Ms = saturation magnetization per mas of material, χ = susceptibility) as a function of the boiling time. The results for the unscaled samples obtained at 30 min boiling time are shown as reference. This table reports the arithmetic averages and the error of the averages with 95% confidence (see text).

Boiling Time (min)	Yield	Particle Size			Organic Content	Magnetic Properties (RT)	
		TEM		XRD			
	*Ρ* (%)	D_TEM_ (nm)	σ_TEM_/D_TEM_ (nm)	D_XRD_ (nm)	Org (%)	Ms (Am^2^/Kg)	χ (Am^2^ Kg^−1^ T^−1^)
5	37 ± 11	13 ± 2	0.33 ± 0.05	13.0 ± 0.2	9.6 ± 0.9	67.5 ± 0.2	940 ± 100
15	48 ± 18	14 ± 1	0.31 ± 0.03	13.3 ± 0.4	9.1 ± 0.4	65.3 ± 1.5	900 ± 100
30	56 ± 16	13 ± 2	0.36 ± 0.01	13.3 ± 0.7	11.1 ± 0.9	63.0 ± 2.1	800 ± 99
60	71 ± 10	16 ± 4	0.29 ± 0.05	13.7 ± 1.0	8.8 ± 0.2	65.7 ± 2.0	790 ± 72
120	61 ± 8	20 ± 1	0.23 ± 0.03	13.5 ± 0.6	11 ± 0.9	67.3 ± 1.8	870 ± 74
Unscaled sample							
30	24 ± 7	14 ± 4	0.27 ± 0.02	12.3 ± 0.4	14 ± 3	66 ± 2	1200 ± 120

**Table 2:** Solvodynamic size average values and SAR at different field conditions measured in nanoparticle suspensions of oleic acid:toluene 1:7 *v*/*v*.

Boiling Time (min)	Solvodynamic Size (0.1 mg Fe/mL)				Magnetic Heating (1 mg Fe/mL)	
					286 kHz 18 kA m^−1^	92 kHz 50 kA m^−1^
	Z_ave_ (nm)	PDI	Number PSD		SAR (W/g Fe)	
			D_SDY_ (nm)	σ (nm)		
5	69 ± 36	0.4 ± 0.2	30 ± 10	8.4 ± 2.7	142 ± 22	74 ± 13
15	77 ± 24	0.6 ± 0.1	27 ± 1.2	6.7 ± 0.3	152 ± 34	80 ± 12
30	104 ± 47	0.7 ± 0.1	28 ± 2.2	6.7 ± 0.4	123 ± 23	71 ± 6
60	128 ± 66	0.7 ± 0.3	28 ± 3	6.6 ± 0.4	129 ± 34	82 ± 16
120	364 ± 12	0.7 ± 0.1	27 ± 0.9	5.6 ± 0.17	121 ± 20	71 ± 11
Unscaled sample						
30	41 ± 2	0.32 ± 0.03	23 ± 0.5	6.0 ± 0.2	96 ± 10	52 ± 8

**Table 3:** Estimation of production cost of 5 g of MNPs by thermal decomposition method under unscaled and scaled conditions.

	Unscaled	Scaled up
Time (h)	27.00	0.50
Reagents cost * (EUR)	279.12	102.18
Energy consumption ** (kWh)	64.26	2.19
Labor cost *** (EUR)	607.50	11.25
Total production cost (EUR)	901.38	113.94

* Reagent cost was estimated using supplier prices from 2021. ** According to Eurostat, the energy price in 2020 was 0.2298 EUR/kWh in Spain (https://ec.europa.eu/eurostat, accessed on 10 April 2021). *** According to Eurostat, the labor cost in 2020 was 22.5 EUR/h in Spain (https://ec.europa.eu/eurostat, accessed on 10 April 2021).

## Data Availability

The raw data used for the present study can be found in the open access repository https://www.zenodo.org/.

## Supplementary Materials

The following are available online at https://www.mdpi.com/article/10.3390/nano11082059/s1: Experimental procedures for the characterization of MNP’s and comparison among the scaled and the standard experiment. Figure S1. Temperature profiles for the scaled-up experiments; Figure S2. Measurements of the successive AMF heating and cooling cycles for selected samples, showing the effects of the progressive destabilization of the colloids; Figure S3. XRD diffraction patterns of all samples; Figure S4. Hysteresis cycles at room temperature of all the samples as measured and corrected for the content of organic matter measured by TG-ATD; Figure S5. Particle size distributions of all the samples and solvodynamic size distributions of their dispersions in toluene determined by PCS in number and in volume; Figure S6. Results of the magnetothermal experiments at 286 kHz 18 kA/m and 92 kHz 50 kA/m for all the samples. Table S1. Average parameters of the temperature profiles for each boiling time, averages of all the experiments, and their repeatability; Table S2. Experimental data of scaled-up samples. Reproducibility r = (1 − σ/$\bar{x}$) · 100. Error of the average: 1.96 · σ/√N (95% confidence). N = 6.

## References

[1] Jung C.W., Jacobs P. (1995). Physical and Chemical-Properties of Superparamagnetic Iron Oxide MR Contrast Agents Ferumoxides, Ferumoxtran, Ferumoxsil. Magn. Reson. Imaging.

[2] Kim B.H., Lee N., Kim H., An K., Park Y.I., Choi Y., Shin K., Lee Y., Kwon S.G., Na H.B. (2011). Large-Scale Synthesis of Uniform and Extremely Small-Sized Iron Oxide Nanoparticles for High-Resolution T-1 Magnetic Resonance Imaging Contrast Agents. J. Am. Chem. Soc..

[3] Pankhurst Q.A., Thanh N.T.K., Jones S.K., Dobson J. (2009). Progress in Applications of Magnetic Nanoparticles in Biomedicine. J. Phys. D Appl. Phys..

[4] Zhang Y., Wu B., Xu H., Liu H., Wang M., He Y., Pan B. (2016). Nanomaterials-Enabled Water and Wastewater Treatment. Nanoimpact.

[5] Gallo-Cordova A., Streitwieser D.A., Morales M.P., Ovejero J.G. (2021). Magnetic Iron Oxide Colloids for Environmental Applications. Colloids—Types, Preparation and Applications.

[6] Gallo-Cordova A., Veintemillas-Verdaguer S., Tartaj P., Mazarío E., Morales M.P. (2021). Engineering Iron Oxide Nanocatalysts by a Microwave-Assisted Polyol Method for the Magnetically Induced Degradation of Organic Pollutants. Nanomaterials.

[7] Shylesh S., Schuenemann V., Thiel W.R. (2010). Magnetically Separable Nanocatalysts: Bridges between Homogeneous and Heterogeneous Catalysis. Angew. Chem. Int. Ed..

[8] Gawande M.B., Branco P.S., Varma R.S. (2013). Nano-Magnetite (Fe_3_O_4_) as a Support for Recyclable Catalysts in the Development of Sustainable Methodologies. Chem. Soc. Rev..

[9] Piñol R., Brites C.D.S., Bustamante R., Martínez A., Silva N.J.O., Murillo J.L., Cases R., Carrey J., Estepa C., Sosa C. (2015). Joining Time-Resolved Thermometry and Magnetic-Induced Heating in a Single Nanoparticle Unveils Intriguing Thermal Properties. ACS Nano.

[10] Rashid H., Mansoor M.A., Haider B., Nasir R., Abd Hamid S.B., Abdulrahman A. (2020). Synthesis and Characterization of Magnetite Nano Particles with High Selectivity Using In-Situ Precipitation Method. Sep. Sci. Technol..

[11] Sun S.H., Zeng H., Robinson D.B., Raoux S., Rice P.M., Wang S.X., Li G.X. (2004). Monodisperse MFe_2_O_4_ (M = Fe, Co, Mn) Nanoparticles. J. Am. Chem. Soc..

[12] Park J., An K.J., Hwang Y.S., Park J.G., Noh H.J., Kim J.Y., Park J.H., Hwang N.M., Hyeon T. (2004). Ultra-Large-Scale Syntheses of Monodisperse Nanocrystals. Nat. Mater..

[13] Laurent S., Forge D., Port M., Roch A., Robic C., Vander Elst L., Muller R.N. (2008). Magnetic Iron Oxide Nanoparticles: Synthesis, Stabilization, Vectorization, Physicochemical Characterizations, and Biological Applications. Chem. Rev..

[14] Roca A.G., Costo R., Rebolledo A.F., Veintemillas-Verdaguer S., Tartaj P. (2009). Progress in the Preparation of Magnetic Nanoparticles for Applications in Biomedicine. Appl. Phys..

[15] Ibarra-Sanchez J.J., Fuentes-Ramirez R., Roca A.G., Morales M.P., Cabrera-Lara L.I. (2013). Key Parameters for Scaling up the Synthesis of Magnetite Nanoparticles in Organic Media: Stirring Rate and Growth Kinetic. Ind. Eng. Chem. Res..

[16] Rubia-Rodríguez I., Santana-Otero A., Spassov S., Tombácz E., Johansson C., De La Presa P., Teran F.J., Morales M.P., Veintemillas-Verdaguer S., Thanh N.T.K. (2021). Whither Magnetic Hyperthermia? A Tentative Roadmap. Materials.

[17] Costo R., Heinke D., Gruettner C., Westphal F., Morales M.P., Veintemillas-Verdaguer S., Gehrke N. (2019). Improving the Reliability of the Iron Concentration Quantification for Iron Oxide Nanoparticle Suspensions: A Two-Institutions Study. Anal. Bioanal. Chem..

[18] Cohen L., Holliday M. (1996). Practical Statistics for Students.

[19] Jensen H., Pedersen J. H., Jorgensen J. E., Skov Pedersen J., Joensen K. D., Iversen S. B., Sogaard E. G. (2006). Determination of size distributions in nanosized powders by TEM, XRD, and SAXS. J. Exp. Nanosci..

[20] Gavilán H., Sánchez E.H., Brollo M.E.F., Asín L., Moerner K.K., Frandsen C., Lázaro F.J., Serna C.J., Veintemillas-Verdaguer S., Morales M.P. (2017). Formation Mechanism of Maghemite Nanoflowers Synthesized by a Polyol-Mediated Process. ACS Omega.

[21] Lartigue L., Hugounenq P., Alloyeau D., Clarke S.P., Lévy M., Bacri J.-C., Bazzi R., Brougham D.F., Wilhelm C., Gazeau F. (2012). Cooperative Organization in Iron Oxide Multi-Core Nanoparticles Potentiates Their Efficiency as Heating Mediators and MRI Contrast Agents. ACS Nano.

[22] Ovejero J.G., Cabrera D., Carrey J., Valdivieso T., Salas G., Teran F.J. (2016). Effects of Inter- and Intra-Aggregate Magnetic Dipolar Interactions on the Magnetic Heating Efficiency of Iron Oxide Nanoparticles. Phys. Chem. Chem. Phys..

[23] Krishnan K.M., Pakhomov A.B., Bao Y., Blomqvist P., Chun Y., Gonzales M., Griffin K., Ji X., Roberts B.K. (2006). Nanomagnetism and Spin Electronics: Materials, Microstructure and Novel Properties. J. Mater. Sci..

[24] Bender P., Fpck J., Frandsen C., Hansen F.M., Balceris C., Ludwig F., Posth O., Bogart L.K., Southern P., Szcerba W. (2018). Relating Magnetic Properties and High Hyperthermia Performance of Iron Oxide Nanoflowers. J. Phys. Chem. C.

[25] Bender P., Honecker D., Fernández Barquín L. (2017). Supraferromagnetic correlations in clusters of magnetic nanoflowers. Appl. Phys. Lett..

[26] Wells J., Ortega D., Steinhoff U., Dutz S., Garaio E., Sandre O., Natividad E., Cruz M., Brero F., Southern P. (2021). Challenges and Recommendations for Magnetic Hyperthermia Characterization Measurements. Int. J. Hyperth..

[27] Salas G., Veintemillas-Verdaguer S., Morales M.P. (2013). Relationship between Physico-Chemical Properties of Magnetic Fluids and Their Heating Capacity. Int. J. Hyperth..

[28] Salas G., Camarero J., Cabrera D., Takacs H., Varela M., Ludwig R., Dähring H., Hilger I., Miranda R., Morales M. (2014). del P.; et al. Modulation of Magnetic Heating via Dipolar Magnetic Interactions in Monodisperse and Crystalline Iron Oxide Nanoparticles. J. Phys. Chem. C.

[29] Harmsen J. (2019). Industrial Process Scale-Up: A Practical Innovation Guide from Idea to Commercial Implementation.

[30] Balakrishnan T., Lee M.-J., Dey J., Choi S.-M. (2019). Sub-Nanometer Scale Size-Control of Iron Oxide Nanoparticles with Drying Time of Iron Oleate. CrystEngComm.

[31] Moon J.-W., Rawn C.J., Rondinone A.J., Love L.J., Roh Y., Everett S.M., Lauf R.J., Phelps T.J. (2010). Large-Scale Production of Magnetic Nanoparticles Using Bacterial Fermentation. J. Ind. Microbiol. Biotechnol..

[32] Simeonidis K., Liébana-Viñas S., Wiedwald U., Ma Z., Li Z.A., Spasova M., Patsia O., Myrovali E., Sakellari D., Tsiaoussis I. (2016). A Versatile Large-Scale and Green Process for Synthesizing Magnetic Nanoparticles with Tunable Magnetic Hyperthermia Features. RSC Adv..

[33] Rui Y.-P., Liang B., Hu F., Xu J., Peng Y.-F., Yin P.-H., Duan Y., Zhang C., Gu H. (2016). Ultra-Large-Scale Production of Ultrasmall Superparamagnetic Iron Oxide Nanoparticles for T-1-Weighted MRI. RSC Adv..

[34] Besenhard M.O., LaGrow A.P., Famiani S., Pucciarelli M., Lettieri P., Thanh N.T.K., Gavriilidis A. (2020). Continuous Production of Iron Oxide Nanoparticles via Fast and Economical High Temperature Synthesis. React. Chem. Eng..

[35] Asimakidou T., Makridis A., Veintemillas-Verdaguer S., Morales M.P., Kellartzis I., Mitrakas M., Vourlias G., Angelakeris M., Simeonidis K. (2020). Continuous Production of Magnetic Iron Oxide Nanocrystals by Oxidative Precipitation. Chem. Eng. J..

