# Supplementary material for: Reproducibility and Scalability of Magnetic Nanoheater Synthesis
Source: Nanomaterials (Basel). 2021 Aug 13;11(8):2059. doi: 10.3390/nano11082059 (PMC8402135; doi:10.3390/nano11082059)
Supplement: Supplementary file 1 [file nanomaterials-11-02059-s001.zip › nanomaterials-1317280-supplementary.pdf]

Supporting Information

Reproducibility and Scalability of Magnetic Nanoheater Synthesis

Jesus G. Ovejero, Alvaro Gallo-Cordova, Alejandro G. Roca, M.P. Morales and Sabino Veintemillas-Verdaguer *

Department of Energy Environment and Health, Instituto de Ciencia de Materiales de Madrid, C.S.I.C., Sor Juana Inés de la Cruz 3, Cantoblanco, ES28049 Madrid, Spain; jesus.g.ovejero@csic.es (J.G.O.); alvaro.gallo@csic.es (A.G.-C.); alejandrogroca@gmail.com (A.G.R.); puerto@icmm.csic.es (M. P.M.)
* Correspondence: sabino@icmm.csic.es

Citation: Ovejero, J.G.; Gallo-Cordova, A.; Roca, A.G.; Morales, M.P.; Veintemillas-Verdaguer, S. Reproducibility and Scalability of Magnetic Nanoheater Synthesis. *Nanomaterials* **2021**, *11*, 2059. https://doi.org/10.3390/nano11082059

Academic Editor: Lenaic Lartigue

Received: 12 July 2021
Accepted: 10 August 2021
Published: date

Publisher's Note: MDPI stays neutral with regard to jurisdictional claims in published maps and institutional affiliations.

Copyright: © 2021 by the authors. Submitted for possible open access publication under the terms and conditions of the Creative Commons Attribution (CC BY) license (http://creativecommons.org/licenses/by/4.0/).

Experimental procedures for the characterization of the MNP's.

TEM observations were made using a JEOL JEM 1011 transmission electron microscope operated at 100 kV with Gatan ES1000Ww camera. Samples were prepared by placing one drop of a dilute particle suspension in toluene on an amorphous carbon-coated copper grid (Carbon coating system Leica EM MED020) and evaporation the toluene at room temperature. In average approximately a thousand of particles were manually counted on 3-5 microphotographs, but in the more polydisperse samples in those more microphotographs were studied given the tendency of smaller and bigger nanoparticles to get together between them during drying.

To obtain dry samples, the dispersion of MNP's in Oleic acid: Toluene 1:7 v/v (3 mL) was precipitated using methanol (10 mL). After the elimination of the supernatant, the MNP's were washed one time with methanol (10 mL) and dried at 50ºC. These samples were used for XRD, TG-ATD and magnetometry.

XRD was performed in a Bruker D8 Advance DAVINCI diffractometer using a zero background holder for the sample (PANanalytical 32mm diameter silicon insert adapted for its use in our diffractometer). The analysis was performed in the range of 20-49º in 2 θ with a scan step size of 0.01º and a scan step time of 1s. The Scherrer size was determined for the three more intense magnetite peaks (220), (311) and (400) using the EVA software.

Thermogravimetric analysis was performed simultaneously in a TG/DTA Q600 TA Instruments. In this work the sample (approximately 10 mg) was heated in Air (100 ml/min) until 800 ºC at a scanning rate 10 ºC/min. As usual the weight loss between 200 and 800 ºC was considered as the organic content of the sample.

Magnetic characterization of the samples was carried out in a vibrating sample magnetometer MagLabVSM (Oxford Instruments) with a maximum field of 5 T. Hysteresis loops of the powder samples were measured at room temperature at a rate of 0.3 T/min. The saturation magnetization was determined by extrapolating to infinite field the magnetization data above 2.5 T using the linear M(H) vs 1/H plot. The magnetic susceptibility was determined as the slope of the M vs H below 0.02T where the dependence of the magnetization and the magnetic field is linear. The mass magnetization was presented in emu/g and the magnetic susceptibility as emu/g T.

The dispersion samples (1 mL) for the measurements of the solvodynamic size D_{SDY} by PCS and SAR by AMF heating measurements were ultrasonically dispersed using an Elmasonic S30 in a glass test tube for 5x2 min and measured immediately.

Comparison among the Scaled and the standard experiment

1-. Energy Flux

We have made measurements of the height (h) that the reactant mixture reaches inside the two spherical reactors of the synthesis and of the internal diameter (d) to calculate the surface A (area of the spherical cap) in contact to the reactant through which the energy is transported.

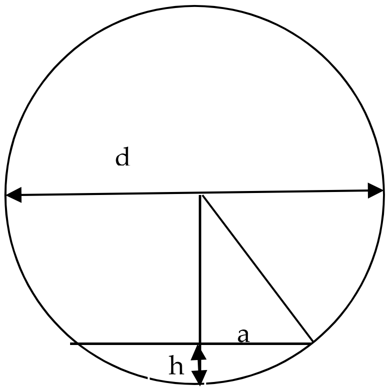

$$a = \sqrt{r^2 - (r - h)^2}$$

$$A = \pi \cdot (a^2 + h^2)$$

The power that is delivered through the spherical cap (w) is approximated by the total power supplied (300 W for the 1 L reactor, and 1300 W for the 10 L reactor) multiplied by the ratio between its surface A and that of the hemisphere S. The energy flow (φ) will finally be obtained by dividing w by A.

**reactor**	**d [cm]**	**h [cm]**	**r [cm]**	**a [cm]**	**A [cm^2]**	**S [cm^2]**	**A/S**	**w [W]**	**Φ [W/cm^2]**
**1 L**	13	1.8	6.5	4.49	73.5	265	0.277	83.1	**1.13**
**10 L**	27.5	4.6	13.75	10.26	397	1187	0.334	434.2	**1.09**

There are no major differences in terms of energy flow, if anything it is less in the scaled experiment.

2-. Energy delivered per gram of reactant.

The energy received per gram of reagent considering 61.31 g of reagent from the 1 L experiment and 1186 g from the 10 L experiment would be 83.1 / 61.31 = **1.36 W / g for the 1 L experiment and 434.2 / 1186 = 0.366 W / g for the one with 10 L**. Therefore, the increase in size of the particles when scaled is not due to having more energy available.

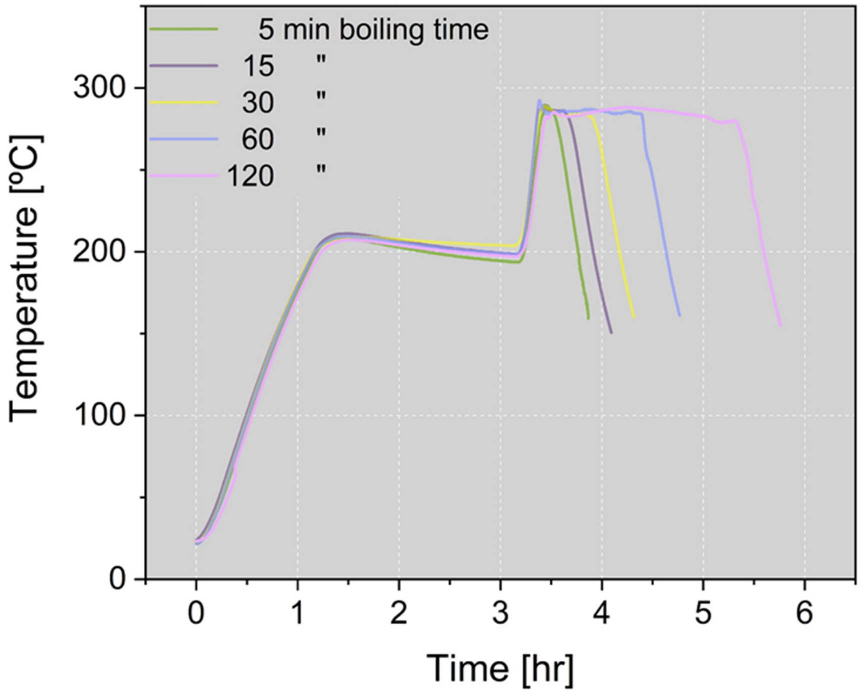

Figure S1 Temperature profiles for the scaled-up experiments

Table S1 Average parameters of the temperature profiles for each boiling time, averages of all the experiments and their repeatability

Boiling time [min]	Ramp 1 25-200 ºC [ºC/min]	Plateau 200ºC		Ramp 2 200-288 ºC [ºC/min]	Boiling temperature	
		$\bar{T}$ [ºC]	σ_T[ºC]		$\bar{T}$ [ºC]	σ_T [ºC]
5	2.76±0.04	201.1±1.4	4.7±0.5	9.04±0.23	285.8±3.8	2.1±0.6
15	2.75±0.03	204.7±3.1	4.3±1.4	9.22±0.12	286.9±2.1	1.9±0.5
30	2.83±0.06	206.1±4.8	2.3±0.8	8.52±0.26	284.8±1.3	2.5±0.4
60	2.80±0.01	203.9±4.7	3.7±1.0	9.06±0.62	285.5±0.6	2.0±1.3
120	2.71±0.07	202.1±1.1	3.5±0.4	9.29±0.39	284.4±1.3	3.5±0.7
**Global Averages**	**2.77±0.04**	**203.6±1.6**	**3.7±0.7**	**9.03±0.24**	**285.5±0.8**	**2.4±0.5**
**σ**	**0.041**	**1.79**	**0.82**	**0.27**	**0.87**	**0.59**
**Reproducibility $(1-\sigma/\bar{x})\cdot 100$**	**98.5%**	**99%**	**77.8%**	**97%**	**99%**	**75.4%**

TABLE S2 Experimental data of scaled-up samples. Reproducibility r= (1-σ/$\bar{x}$) ·100. Error of the average: 1.96·σ/√N (95% confidence) N=6

	Exp.	Yield [%]	TEM D_{TEM} [nm]	TEM σ_{TEM} [nm]	TEM σ_{TEM}/D_{TEM}	DRX Scher-rer's size D_{DRX} [nm]	PCS D_{SDY} 0.1 mgFe/mL Z_{ave} [nm]	PCS D_{SDY} 0.1 mgFe/mL PDI	TG-ATD Or-ganic wt%	Magnetic properties at room temperature Ms emu/g	Magnetic properties at room temperature χ emu/gT	MAGNETIC HIPERTHERMIA 1mgFe/mL 286 kHz 18 kAm^{-1} SAR [W/gFe]	MAGNETIC HIPERTHERMIA 1mgFe/mL 92 kHz 50 kAm^{-1} SAR [W/gFe]
5 min	OR12	36	9.0	2.5	0.28	13.2±0.6	170	0.8	8.35	66.43±0.19	847±51	100	58
	OR13	35	12.8	3.5	0.27	12.6±1.8	43	0.3	9.40	65.39±0.51	794±46	109	85
	OR14	13	15.2	6.0	0.39	13.1±0.8	56	0.4	11.28	64.44±0.45	1004±62	159	101
	OR15	61	11.5	4.7	0.41	13.0±1.2	58	0.4	8.87	73.15±1.1	1188±69	164	57
	OR16	38	13.6	4.5	0.33	13.2±1.8	51	0.3	8.85	67.74±0.43	923±42	155	80
	OR30	40	15.3	4.1	0.27	12.7±0.5	38	0.2	10.94	67.76±0.27	873±47	163	62
$\bar{x}$		**37±11**	**13±2**	**4.2±0.9**	**0.33±0.05**	**13.0±0.2**	**69±36**	**0.4±0.2**	**9.6±0.9**	**67.5±0.2**	**938±103**	**142±22**	**74±13**
σ		**14**	**2.2**	**1.1**	**0.057**	**0.23**	**45**	**0.2**	**1.10**	**2.8**	**129**	**27**	**16.2**
r [%]		**62**	**83**	**74**	**83**	**98**	**35**	**50**	**88**	**96**	**86**	**81**	**78**
15 min	OR17	71	15.5	4.2	0.27	13.8±1.1	62	0.5	9.15	65.52±0.16	1105±60	229	100
	OR18	82	12.9	4.0	0.31	13.1±1.3	63	0.6	8.82	65.91±0.25	923±46	177	86
	OR19	40	15.7	5.6	0.36	14.0±1.1	77	0.8	9.90	64.54±0.22	709±30	155	81
	OR20	30	13.1	4.7	0.36	12.9±1.2	52	0.4	8.34	64.79±0.11	813±49	128	91
	OR21	21	13.6	4.0	0.29	12.7±0.7	66	0.5	8.82	62.37±0.13	998±57	113	70
	OR31	44	13.4	3.8	0.29	13.2±1.9	144	0.5	9.42	68.49±0.06	885±41	109	54
$\bar{x}$		**48±18**	**14±0.8**	**4.4±0.5**	**0.31±0.03**	**13.3±0.4**	**77±24**	**0.6±0.1**	**9.1±0.4**	**65.27±1.5**	**906±101**	**152±34**	**80±12**
σ		**22**	**1.1**	**0.6**	**0.035**	**0.47**	**30**	**0.12**	**0.50**	**1.82**	**127**	**42**	**15**
r [%]		**54**	**92**	**86**	**89**	**96**	**61**	**80**	**95**	**97**	**86**	**72**	**81**
30 min	OR1	57	11.5	3.9	0.34	13.1±1.7	220	0.8	10.91	59.59±0.18	665±24	105	70
	OR2	51	16.7	6.0	0.36	13.6±1.4	108	0.8	11.35	62.78±0.24	817±31	150	77
	OR3	81	10.4	4.1	0.39	11.5±2.5	70	0.8	15.01	60.01±0.09	738±39	98.2	61
	OR5	55	10.0	3.4	0.34	14±0.57	123	0.6	7.33	63.69±0.13	760±26	111	67
	OR22	69	15.9	5.8	0.36	13.5±1.4	40	0.3	8.98	65.49±0.23	824±45	100	69
	OR23	21	14	5	0.36	14.3±1.4	61	0.6	10.55	66.67±0.34	1063±74	175	81
$\bar{x}$		**56±16**	**13±2**	**4.7±0.9**	**0.36±0.01**	**13.3±0.7**	**104±47**	**0.7±0.1**	**11±1.9**	**63.04±2.1**	**811±99**	**123±23**	**71±6**
σ		**20**	**2.8**	**1.1**	**0.018**	**0.90**	**59**	**0.18**	**2.36**	**2.60**	**124**	**29**	**7**
r [%]		**64**	**78**	**76**	**95**	**93**	**43**	**74**	**78**	**96**	**85**	**76**	**90**
60 min	OR6	67	10.0	3.2	0.32	14.3±2.5	159	1	7.67	69.06±0.16	784±48	169	77
	OR7	66	17.6	4.3	0.24	11.5±0.7	41	0.3	11.69	62.08±0.22	712±20	103	59
	OR8	87	19.8	5.9	0.30	15.6±2.4	80	0.8	6.72	68.44±0.19	819±34	74	68
	OR9	80	14.1	4.1	0.28	13.0±0.8	36	0.2	9.36	63.15±0.27	628±20	90	87

	OR10	51	22.0	4.4	0.20	13.7±1.6	192	1	8.56	66.32±0.31	902±47	186	106
	OR11	73	13.5	5.2	0.39	14.1±1.4	261	1	8.54	65.19±0.21	851±60	151	94
$\bar{x}$		**71±10**	**16±4**	**4.5±0.7**	**0.29±0.05**	**13.7±1.0**	**128±66**	**0.7±0.3**	**8.8±1.2**	**65.71±2.0**	**783±72**	**129±34**	**82±16**
σ		**12.5**	**4.5**	**0.9**	**0.066**	**1.26**	**83**	**0.34**	**1.55**	**2.55**	**90**	**42**	**15.8**
**r [%]**		**82**	**72**	**80**	**77**	**90**	**35**	**51**	**82**	**96**	**89**	**67**	**81**
**120 min**	OR24	59	18.2	4.4	0.24	14.0±0.5	616	0.8	12.79	69.18±0.12	1007±51	169	96
	OR25	43	19.0	5.7	0.30	13.6±0.9	282	0.8	12.48	65.71±0.10	887±45	137	66
	OR26	56	18.1	3.9	0.22	13.7±0.8	263	0.7	11.24	66.69±0.10	689±16	108	82
	OR27	74	18.1	4.2	0.23	13.6±0.3	207	0.7	10.41	69.01±0.25	869±9	108	57
	OR28	67	22.8	4.3	0.19	14.1±0.4	533	0.3	9.97	69.79±0.08	857±33	107	62
	OR29	66	21.1	3.7	0.18	12.0±1.3	284	0.7	11.08	63.53±0.01	884±44	96	63
$\bar{x}$		**61±8**	**20±1**	**4.4±0.5**	**0.23±0.03**	**13.5±0.6**	**364±121**	**0.7±0.1**	**11±0.9**	**67.3±1.8**	**866±74**	**121±20**	**71±11**
σ		**9.8**	**1.8**	**0.6**	**0.039**	**0.697**	**152**	**0.17**	**1.11**	**2.22**	**93**	**25**	**14**
**r [%]**		**84**	**91**	**86**	**83**	**95**	**58**	**76**	**90**	**97**	**89**	**79**	**80**

Table S2. Unscaled 1/20 samples for reference

	**Exp.**	**Yield [%]**	**TEM**			**DRX Scherrer's size**	**PCS D_{SDY} 0.1mgFe/mL**		**TG-ATD**	**Magnetic properties at room temperature**		**MAGNETIC HIPERTHERMIA 1mgFe/mL**	
			**D_{TEM} [nm]**	**σ_{TEM} [nm]**	**σ_{TEM}/D_{TEM}**	**D_{DRX} [nm]**	**Z_{ave} [nm]**	**PDI**	**Organic wt%**	**Ms emu/g**	**γ emu/gT**	**286 kHz 18 kAm^{-1}**	**92 kHz 50 kAm^{-1}**
												**SAR [W/gFe]**	
**30 min**	ORD1	43.6	14.1	4.3	0.30	12±0.5	37	0.26	12.03	66.90±0.36	1120±68	98.2	71.7
	ORD2	23.3	13.3	3.1	0.23	12±0.9	40	0.34	12.20	67.06±0.16	945±44	69.8	42.2
	ORD3	19.7	15.0	3.8	0.26	13±1.4	44	0.37	21.21	60.04±0.11	863±54	98.1	52.7
	ORD4	18.5	14.1	3.8	0.27	12±0.7	~~646~~	~~0.49~~	9.66	68.21±0.11	952±59	111	42.1
	ORD5	19.8	13.9	3.8	0.28	12±1	41	0.32	13.52	65.92±0.06	870±41	94.3	47.5
	ORD6	21.4	13.8	4.0	0.29	13±0.9	42	0.32	12.21	68.03±0.18	1261±94	109	55.3
		**24.4±7**	**14.0±0.4**	**3.8±0.3**	**0.27±0.02**	**12.3±0.4**	**41±2**	**0.32±0.03**	**13.5±2.9**	**66.03±2.2**	**1002±114**	**96±10**	**52±8**
σ		**8.72**	**0.51**	**0.36**	**0.022**	**0.47**	**2.31**	**0.036**	**3.64**	**2.78**	**143**	**13**	**10**
**r [%]**		**64**	**96**	**91**	**92**	**96**	**94**	**89**	**73**	**96**	**86**	**87**	**81**

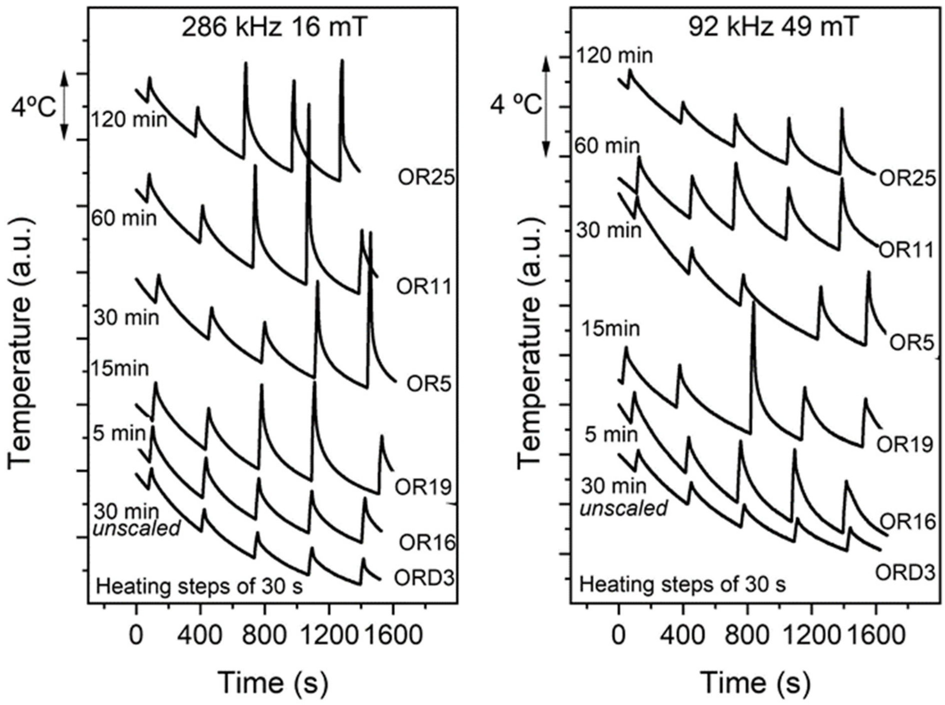

Figure S2 Measurements of the successive AMF heating and cooling cycles for selected samples showing the effects of the progressive destabilization of the colloids.

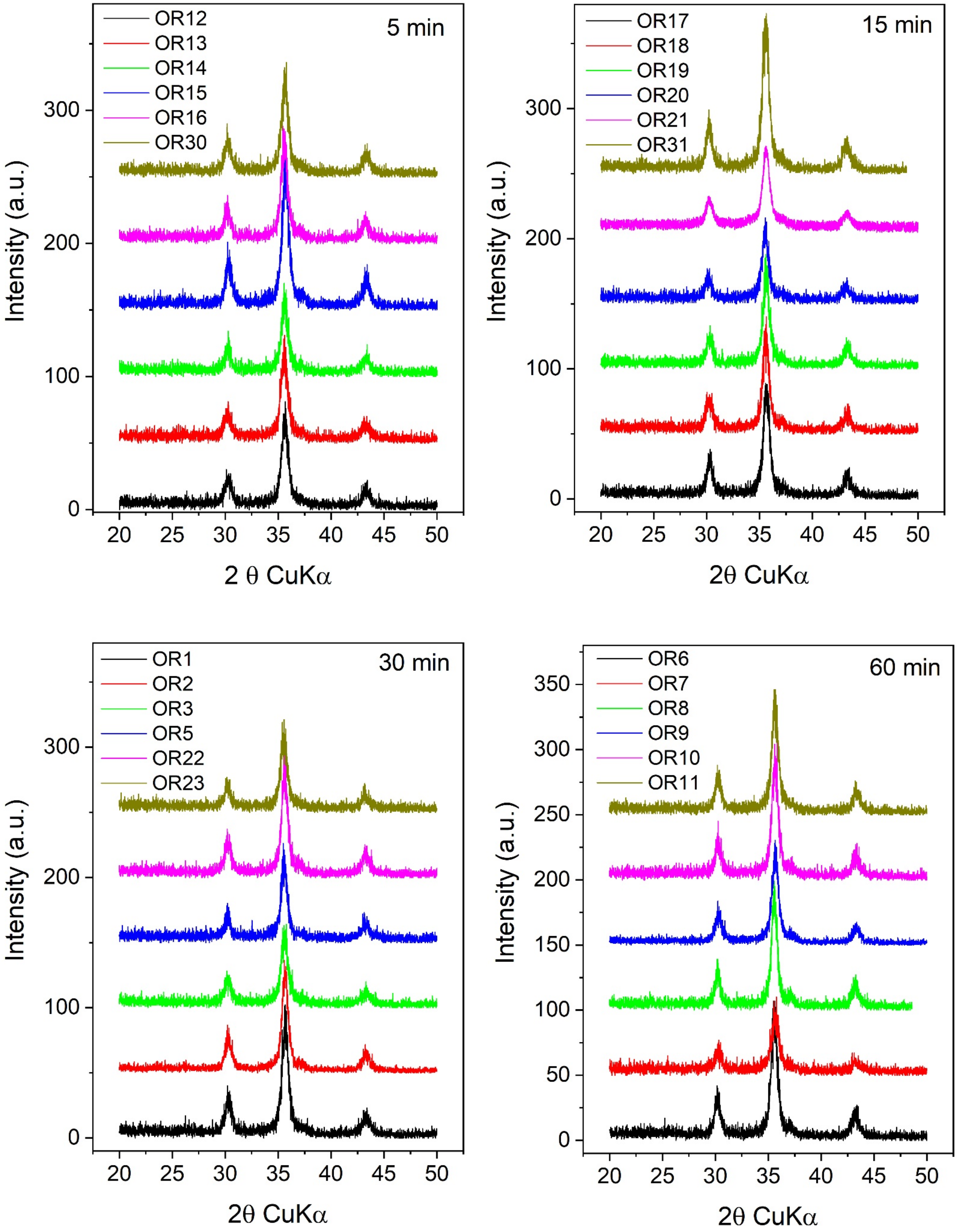

5 min
OR12
OR13
OR14
OR15
OR16
OR30
Intensity (a.u.)
2 θ CuKα
15 min
OR17
OR18
OR19
OR20
OR21
OR31
2θ CuKα
30 min
OR1
OR2
OR3
OR5
OR22
OR23
60 min
OR6
OR7
OR8
OR9
OR10
OR11

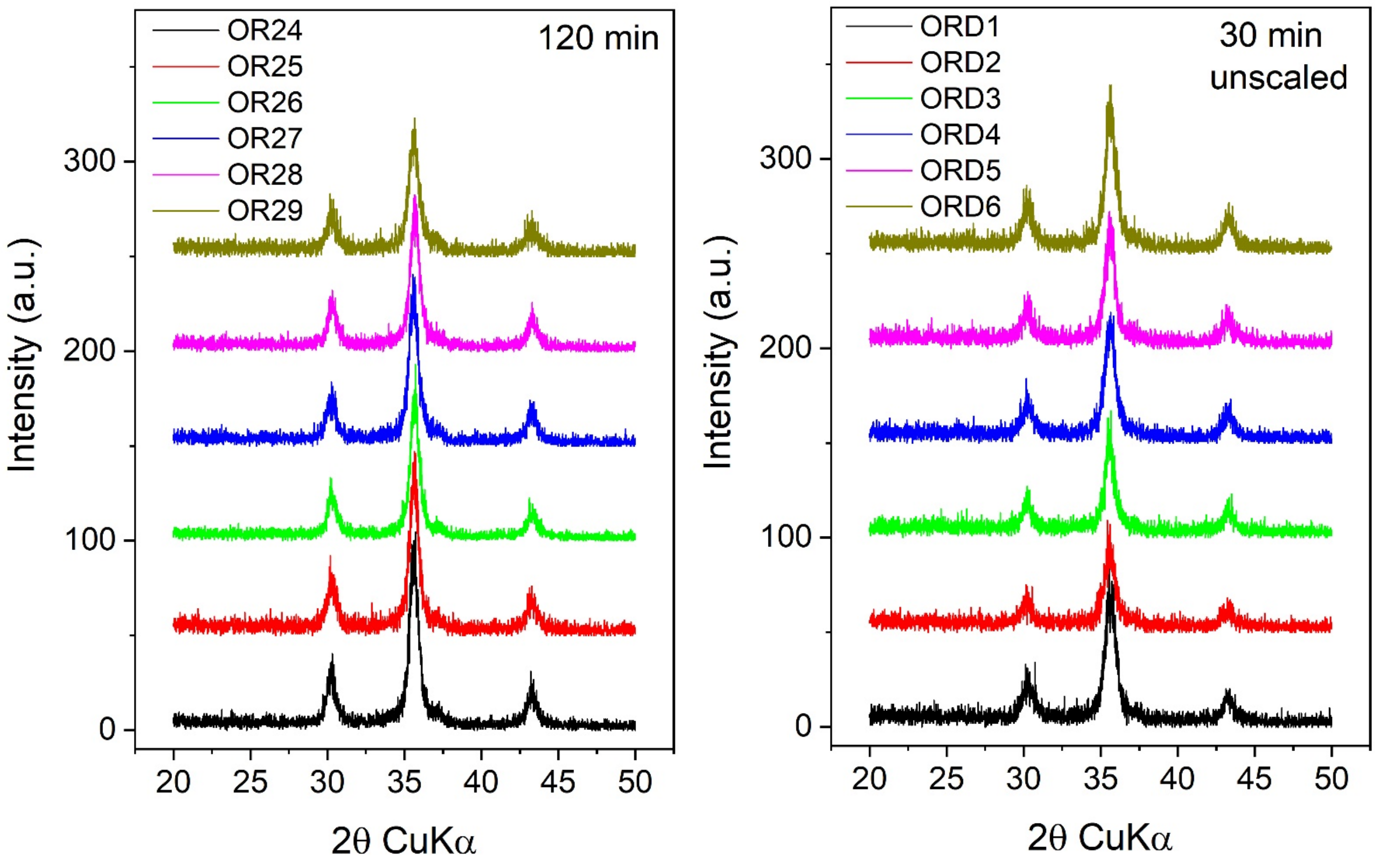

Figure S3: XRD diffraction patterns of all samples.

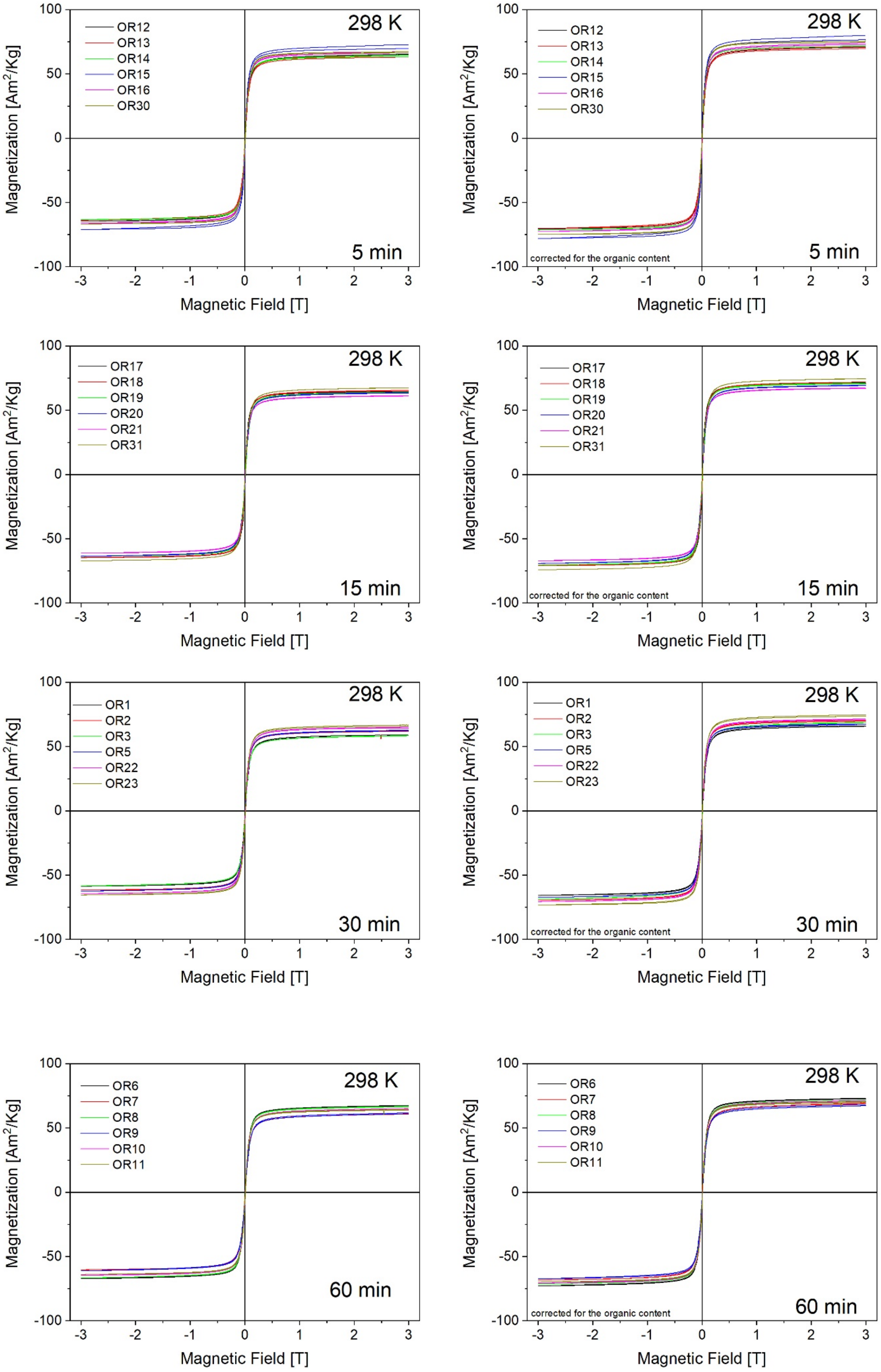

298 K
5 min
15 min
30 min
60 min
corrected for the organic content
Magnetization [Am²/Kg]
Magnetic Field [T]
OR12
OR13
OR14
OR15
OR16
OR30
OR17
OR18
OR19
OR20
OR21
OR31
OR1
OR2
OR3
OR5
OR22
OR23
OR6
OR7
OR8
OR9
OR10
OR11
100
50
0
-50
-100
-3
-2
-1
0
1
2
3

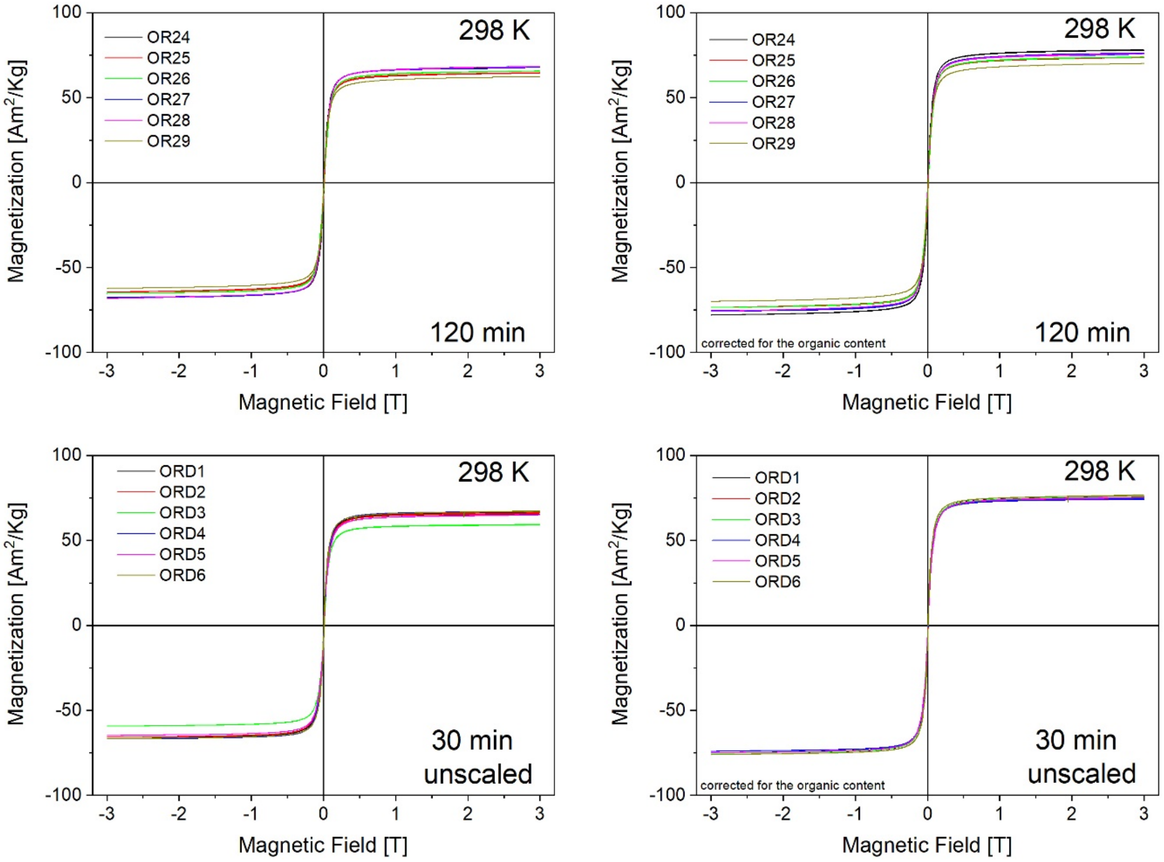

Figure S4 Hysteresis cycles at room temperature of all the samples as measured (left) and corrected for the content of organic matter measured by TG-ATD (right).

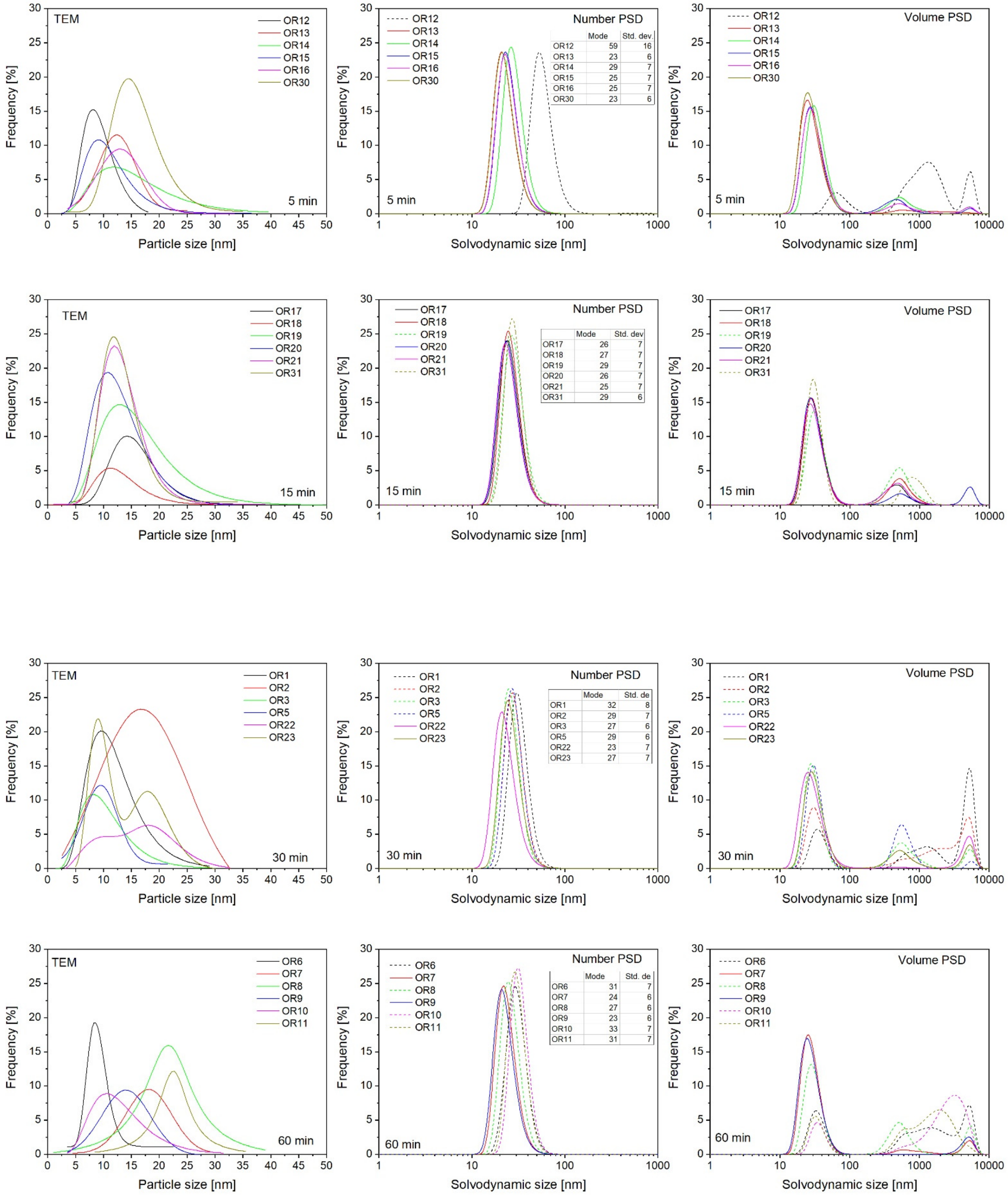
TEM
OR12
OR13
OR14
OR15
OR16
OR30
5 min
Frequency [%]
Particle size [nm]
Number PSD
Mode
Std. dev.
OR12 59 16
OR13 23 6
OR14 29 7
OR15 25 7
OR16 25 7
OR30 23 6
Solvodynamic size [nm]
Volume PSD
TEM
OR17
OR18
OR19
OR20
OR21
OR31
15 min
Number PSD
Mode
Std. dev
OR17 26 7
OR18 27 7
OR19 29 7
OR20 26 7
OR21 25 7
OR31 29 6
Volume PSD
TEM
OR1
OR2
OR3
OR5
OR22
OR23
30 min
Number PSD
Mode
Std. de
OR1 32 8
OR2 29 7
OR3 27 6
OR5 29 6
OR22 23 7
OR23 27 7
Volume PSD
TEM
OR6
OR7
OR8
OR9
OR10
OR11
60 min
Number PSD
Mode
Std. de
OR6 31 7
OR7 24 6
OR8 27 6
OR9 23 6
OR10 33 7
OR11 31 7
Volume PSD

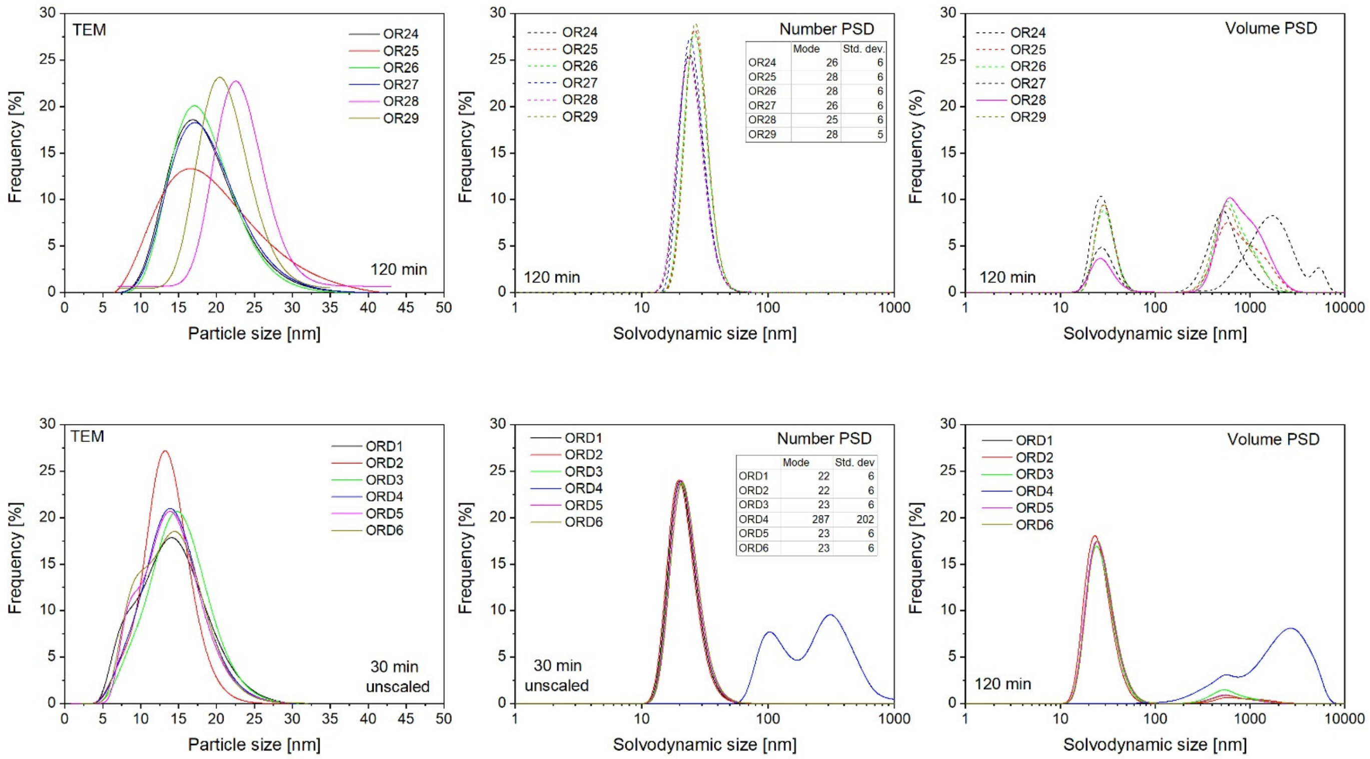

Figure S5 Particle size distributions of all the samples (left) and solvodynamic size distributions of their dispersions in toluene determined by PCS in number (center) and in volume (right). Polydisperse samples unable to be treated by the Contin algorithm are pictured with dash lines. The dispersion of the sample ORD4 was excluded from the statistical analysis.

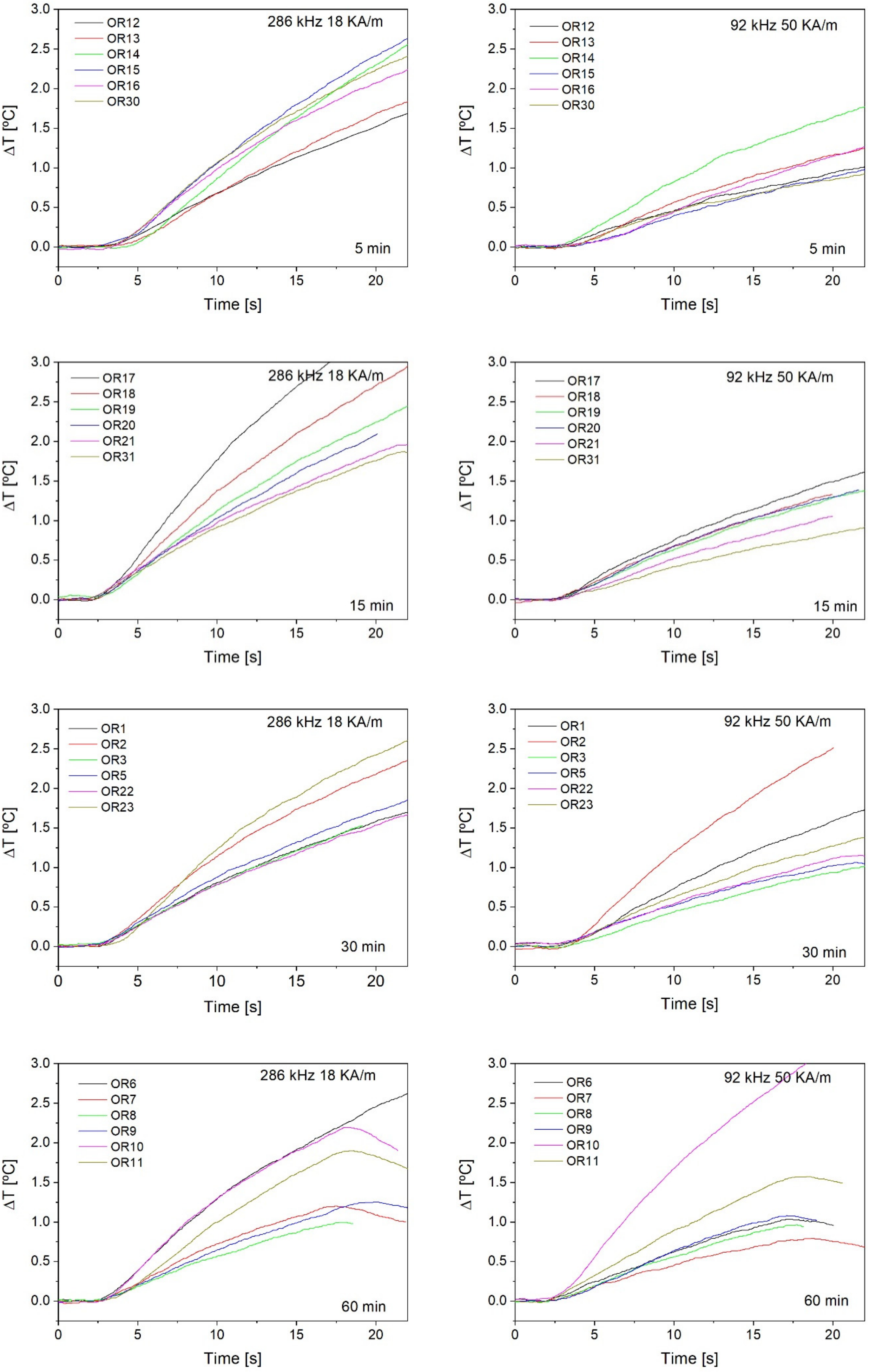
286 kHz 18 KA/m
92 kHz 50 KA/m
OR12
OR13
OR14
OR15
OR16
OR30
5 min
OR17
OR18
OR19
OR20
OR21
OR31
15 min
OR1
OR2
OR3
OR5
OR22
OR23
30 min
OR6
OR7
OR8
OR9
OR10
OR11
60 min
ΔT [ºC]
Time [s]

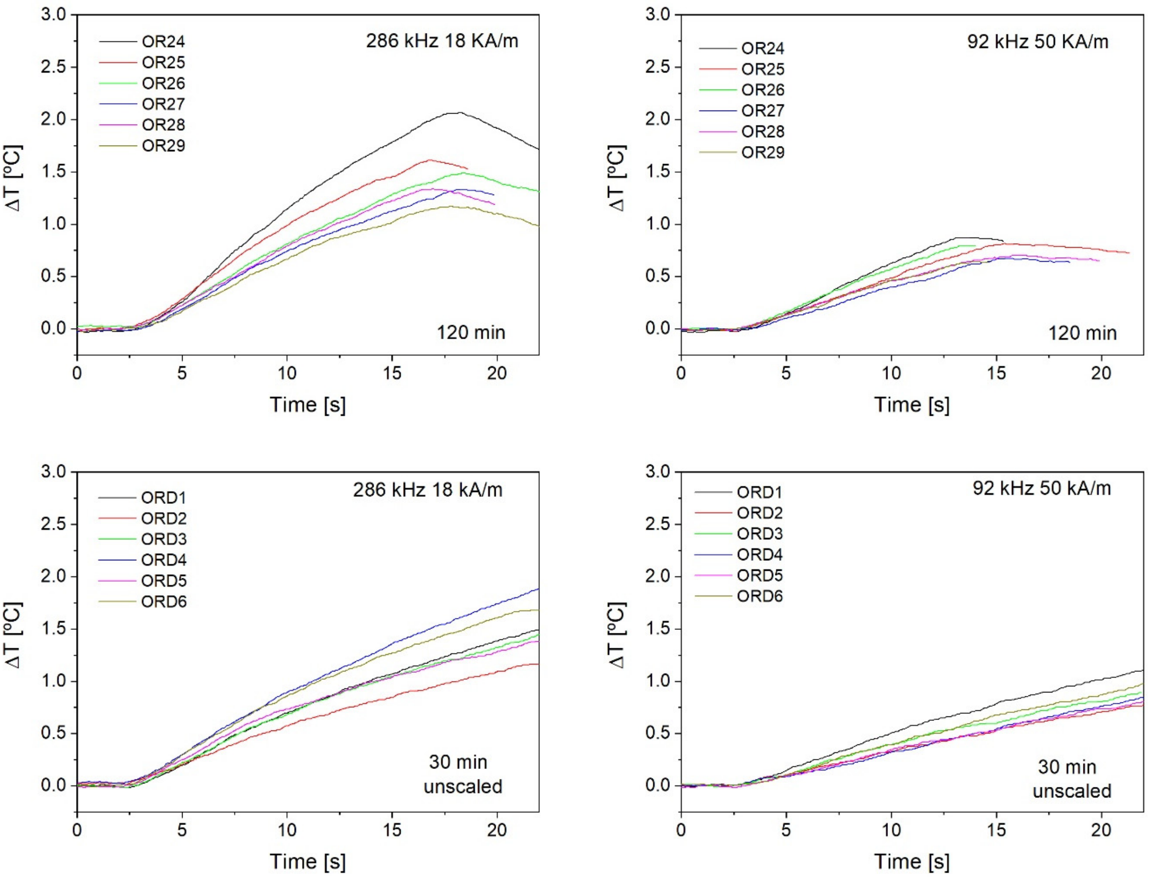

Figure S6: Results of the magnetothermal experiments at 286 kHz 18 kA/m and 92 kHz 50 kA/m for all the samples. The samples were dispersed in oleic acid: toluene 1:7 v/v at 1 mg Fe/ml.
